# Raman spectroscopy and group and basis-restricted non negative matrix factorisation identifies radiation induced metabolic changes in human cancer cells

**DOI:** 10.1038/s41598-021-83343-5

**Published:** 2021-02-16

**Authors:** Kirsty Milligan, Xinchen Deng, Phillip Shreeves, Ramie Ali-Adeeb, Quinn Matthews, Alexandre Brolo, Julian J. Lum, Jeffrey L. Andrews, Andrew Jirasek

**Affiliations:** 1 Department of Physics, The University of British Columbia, Kelowna, Canada; 2 Department of Statistics, The University of British Columbia, Kelowna, Canada; 3 BC Cancer, Centre for the North, Prince George, Canada; 4 Department of Chemistry, University of Victoria, Victoria, Canada; 5 Department of Biochemistry and Microbiology, University of Victoria, Victoria, Canada; 6 Trev and Joyce Deeley Research Centre, BC Cancer, Victoria, Canada

**Keywords:** Cell biology, Biomarkers, Mathematics and computing, Cancer, Breast cancer, Cancer metabolism, Cancer therapy, Lung cancer, Physics, Applied physics, Analytical chemistry

## Abstract

This work combines single cell Raman spectroscopy (RS) with group and basis restricted non-negative matrix factorisation (GBR-NMF) to identify individual biochemical changes associated with radiation exposure in three human cancer cell lines. The cell lines analysed were derived from lung (H460), breast (MCF7) and prostate (LNCaP) tissue and are known to display varying degrees of radio sensitivity due to the inherent properties of each cell type. The GBR-NMF approach involves the deconstruction of Raman spectra into component biochemical bases using a library of Raman spectra of known biochemicals present in the cells. Subsequently, scores are obtained on each of these bases which can be directly correlated with the contribution of each chemical to the overall Raman spectrum. We validated GBR-NMF through the correlation of GBR-NMF-derived glycogen scores with scores that were previously observed using principal component analysis (PCA). Phosphatidylcholine, glucose, arginine and asparagine showed a distinct differential score pattern between radio-resistant and radio-sensitive cell types. In summary, the GBR-NMF approach allows for the monitoring of individual biochemical radiation-response dynamics previously unattainable with more traditional PCA-based approaches.

## Introduction

It is estimated that radiation therapy (RT) is used to treat $\sim 50\%$ of cancers worldwide^1^ and is currently the most cost-effective treatment for most types of cancers^2^. Altered cellular metabolism is a hallmark of tumour cells^3,4^ and a contributing factor in tumour cell resistance to both radiation therapy and anti-cancer drugs. This has created significant interest in the development of more personalised and effective methods of radiation treatment delivery. To develop treatment plans which are specific to tumour type and environment, a better understanding of the radiation-induced biochemical changes which take place within the tumour environment is required.

Raman spectroscopy (RS) is a non-invasive, label-free optical spectroscopic technique which can be used to obtain spectral information pertaining to the biochemicals present in live cells both pre and post radiation treatment. As a result, specific radiation induced responses can be monitored within a given cell population and potential therapeutic targets identified^5,6^. In this study, three human tumour cell lines were irradiated and analysed using RS. The cells were derived from human lung (H460), breast (MCF7) and prostate (LNCaP) tumour cell lines. Previous studies have shown that both MCF7 and H460 cells display a radiation-induced accumulation of glycogen which is correlated with radiation resistance^7^. The metabolism of glycogen involves an array of complex signalling pathways, many of which can be directly related to tumour progression^8–12^. Increase in glycogen content within tumour cells post radiation treatment is thought to provide metabolic precursors which protect against hypoxia and other forms of stress^13^.

Identifying metabolites which are associated with tumour progression and treatment resistance enable the development of more personalised, cancer-specific treatment options. Matthews et al.^7^ identified metformin, a drug widely used to treat type-2 diabetes, as a potential candidate for use in combination with radiation therapy. Specifically, 5 mM metformin was shown to reverse the glycogen accumulation observed in MCF7 breast cancer cells after treatment with small doses of radiation. As a result, the previously radio-resistant cells displayed enhanced levels of radio-sensitivity. These results support the possibility that manipulation of metabolic pathways could provide a therapeutic strategy to enhance sensitivity to RT.

In much of the literature exploring radiation response using RS, principal component analysis (PCA) has been used as the primary data analytic tool. The main drawback of using dimensionality reducing techniques such as PCA in combination with RS is the difficulty often encountered when interpreting the relationship between positive and negative attributes of principal components and how these can be correlated with individual biochemical responses within cells and tumours. Aside from the drawbacks of PCA allowing components to assume negative values, which is an incorrect representation of spectroscopic data, there is also a constraint of orthogonality within the principal components, which can restrict our interpretation of the features which are responsible for the variance within the dataset. Additionally, principal components are often combinations of spectral features relating to multiple cellular bio-components which can confound identification of specific biochemicals^14^.

**Figure 1:**
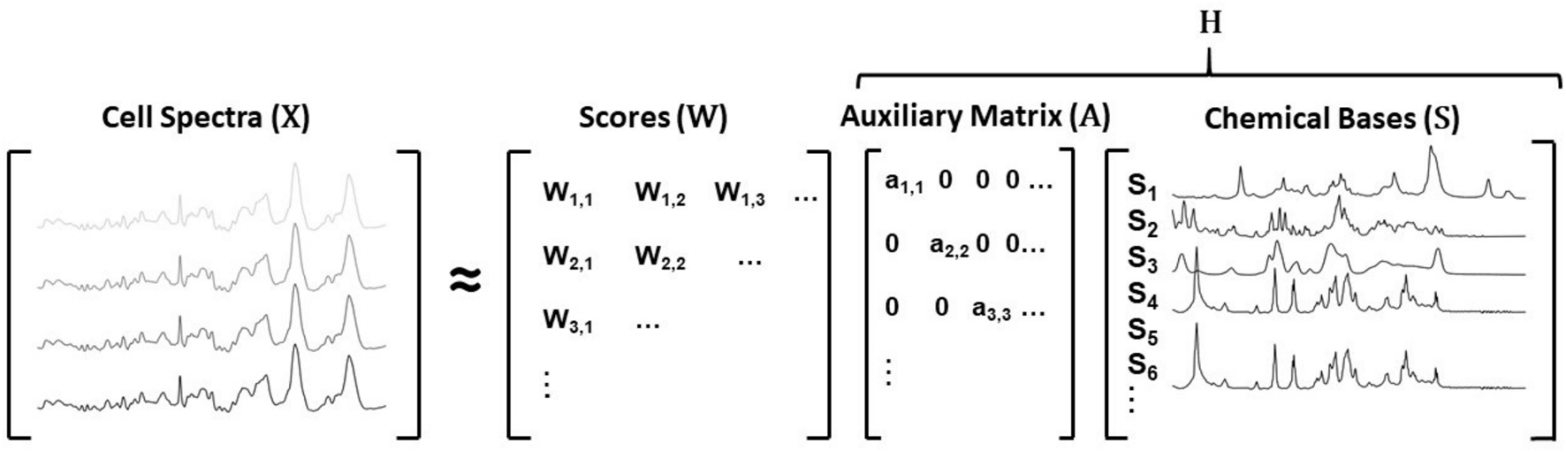
Schematic depicting the decomposition of the matrix ${\mathbb {X}}$ (cell spectra) into lower rank non negative matrices ${\mathbb {W}}$ (scores), ${\mathbb {A}}$ (auxiliary) and ${\mathbb {S}}$ (chemical bases). This method is semi-supervised when compared with traditional NMF as matrix ${\mathbb {H}}$ is further decomposed into two matrices ${\mathbb {A}}$ and ${\mathbb {S}}$, where A is an auxiliary matrix used to scale the data such that the mean score on each factor equates to 1 and ${\mathbb {S}}$ is a matrix containing Raman spectra of known biochemicals which can be fully or partially pre-specified.

We therefore report an alternate approach, wherein a variant of non-negative matrix factorisation (NMF) is used to identify radiation induced responses specific to a known library of chemical bases. NMF was originally developed by Lee and Seung^15^ to provide an additive, parts-based representation of a non-negative data matrix. A data matrix ${\mathbb {X}}$ can be decomposed into two lower rank non-negative matrices ${\mathbb {W}}$ and ${\mathbb {H}}$, where ${\mathbb {W}}$ can be referred to as scores on the factors found in ${\mathbb {H}}$. In group and basis restricted NMF (GBR-NMF)^16^, the ${\mathbb {H}}$ matrix is further decomposed into two non-negative matrices ${\mathbb {A}}$ and ${\mathbb {S}}$, where ${\mathbb {A}}$ is a diagonal matrix providing scaling for the factors either found or pre-specified in ${\mathbb {S}}$. In our implementation of GBR-NMF, the observations input to the model ${\mathbb {X}}$ were Raman spectra, each collected from the three cell lines aforementioned at various time points and doses of ionising radiation. Each spectrum then has a non-negative score ${\mathbb {W}}$ estimated on each of the factors in ${\mathbb {S}}$, which in this case includes individual biochemical bases along with an unconstrained factor that can be estimated from the data. This model differs from conventional NMF in that the factors are partially constrained by the biochemical bases. That is, all factors but one are constrained by biochemical profile, with the remaining profile being allowed to be estimated from the data—in other words, be unconstrained. The ${\mathbb {A}}$ matrix is an auxiliary matrix used to scale the data such that the mean score for each factor equates to 1. In this instance, the ${\mathbb {S}}$ matrix consisted of Raman spectra of 30 biochemicals (listed in Table S1, spectra are shown in figures S1 and S2) as constrained factors alongside one unconstrained factor estimated from the data. The scores on each of the chemical bases were monitored as a result of radiation dose and time subsequent to exposure. A schematic representation of the model is shown in Fig. 1.

In this model, a library consisting of 30 Raman spectra of the biochemicals (listed in S1) were utilised as the biochemical bases. The 30 biochemicals chosen represent four classes of biochemicals present in cells—nucleic acids, proteins, lipids and carbohydrates^17^. Biochemicals which are not represented by the constrained bases, should be accounted for in the unconstrained component of the model. Spectra can be added or removed to the model, dependent on the biochemical make up of the samples and components which may be of particular interest. Raman spectra for each cell line, acquired subsequent to treatment with clinically relevant doses of ionising radiation (2–10 Gy) and 0 Gy controls, on days 1–3 post treatment were the input data. The GBR-NMF model then produced scores on each of the 30 biochemical bases spectra, for each cell type, under each condition described above. The scores obtained are similar in nature to PCA scores, which correlate how much a sample spectrum can be described by the positive and negative features of the principal component output by the model^18,19^. However, as GBR-NMF is a semi-supervised approach, the scores obtained correlate directly with the contributions of the biochemical bases to the sample spectrum. These scores allow us to identify biochemical score patterns (expression dynamics) with respect to the conditions under which the initial input data was obtained—for example: cell type, radiation dose, time post radiation treatment.

The major benefit of using the GBR-NMF approach over other data analysis techniques such as PCA, is the capacity to identify, with reasonable confidence, individual biochemicals which may be involved in metabolic pathways relating to radiation resistance. The ability to identify radiation induced response profiles within various cell types opens up a number of new treatment pathways that could be exploited in order to both increase the radio-sensitivity of the tumour cells as well as the possibility to explore new, combination therapies. Combining radiation therapy with, for example, the addition of radio-sensitising agents in order to induce anti-tumour mechanisms, may result in a more effective treatment.

The GBR-NMF model produces scores on biochemicals which can also be used in conjunction with other machine learning techniques. Random forest^20^ (RF) is a supervised learning method which can be used for classification or regression. Random forests are generated by constructing a large number of decision trees, which when their predictions are systematically combined have strong predictive power^21^. An advantage of this type of machine learning is that it allows us to measure the importance of the features in the model based on the classifications provided and the success of the classification^22,23^. There are two main ways that this is often measured in classification RF: mean decrease accuracy (MDA) and mean node impurity (MNI). MDA measures the decrease in accuracy of the model when the observations on the variable in question are randomly permuted. The greater the decrease in accuracy due to the permuting of each variable, the greater the importance of that variable in correctly classifying the data. In our applications, the MDA and MNI measures provided very similar results, and so we will only discuss MDA in this work. Combining GBR-NMF with RF allows us to discern the important biochemicals which contribute to the classification of the three cell types, H460, MCF7 and LNCaP, as radio-sensitive or radio-resistant.

## Results

### Comparison of PCA and GBR-NMF score trends with radiation exposure

**Figure 2:**
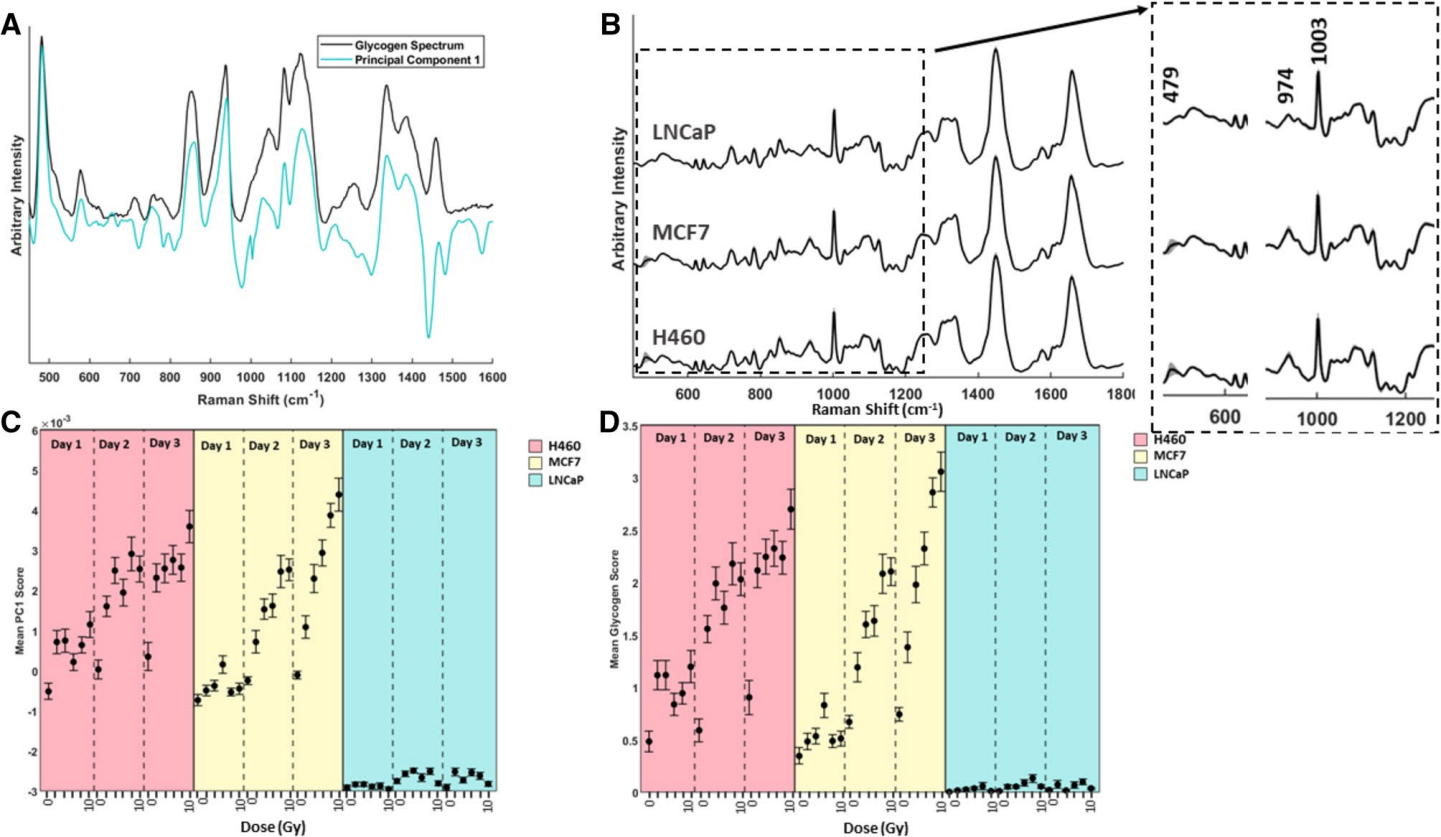
**(A)** RS of pure glycogen (black) overlaid with principal component 1 (green). **(B)** Average RS obtained from all days and doses including unirradiated controls for H460 cells, MCF7 cells and LNCaP cells. Shadow spectrum represents $+/-$ 1 standard deviation at each wavenumber. The greatest deviation in H460 and MCF7 cells is visible at $479 \,\hbox {cm}^{-1}$, which can be attributed to the change in glycogen content with respect to day and dose of radiation received. Inset spectra highlight the larger standard deviation (shadow spectrum) from the mean at $479 \,\hbox {cm}^{-1}$, $974\, \hbox {cm}^{-1}$ and $1003 \,\hbox {cm}^{-1}$ for MCF7 and H460 cells compared with a smaller standard deviation observed in LNCaP cells. **(C)** Mean PC1 scores for H460 cells (pink), MCF7 cells (yellow) and LNCaP cells (blue) for doses 2–10 Gy on days 1–3 post irradiation. Error bars represent $+/-$ 1 standard error. **(D)** Mean glycogen scores for H460 cells (pink), MCF7 cells (yellow) and LNCaP cells (blue) for doses 2–10 Gy on days 1–3 post irradiation, obtained using the GBR-NMF model. Error bars represent $+/-$ 1 standard error.

One major advantage of using a semi-supervised NMF approach is the ability to monitor the response of specific biochemicals as a result of radiation exposure. We applied GBR-NMF to examine two radio-resistant human tumour cell types, derived from oestrogen receptor positive breast cell line (MCF7) and non-small cell lung tumour cell line (H460). A human prostate tumour cell line (LNCaP) was also examined as this cell line is known to be radio-sensitive^6^. Each cell line was exposed to single fractions of 0, 2, 4, 6, 8 and 10 Gy radiation. Single cell Raman spectra were obtained on days 1, 2 and 3 post-irradiation, as described by Matthews et al.^7^ Prior to GBR-NMF, PCA was carried out on the entire data set, wherein the first principal component (PC1) very closely resembled that of a pure glycogen spectrum (Fig. 2A)^7^.

The average spectrum for each cell line including all doses and days is shown in Fig 2B. The standard deviation at each wavenumber is highlighted in each spectrum, which clearly shows a greater deviation from the mean for both MCF7 and H460 cells at $479\, \hbox {cm}^{-1}$, correlating with increased glycogen content. This large deviation was not observed in LNCaP cells. The inset spectra in Fig. 4B show various other spectral regions where there was a noticeable deviation from the mean across the dataset, such as $974 \,\hbox {cm}^{-1}$ and $1003 \,\hbox {cm}^{-1}$ in both MCF7 and H460 cell types. Matthews et al.^7^ have previously shown that both radio-resistant cell lines (MCF7 and H460) show a trend pertaining to increased glycogen accumulation with respect to radiation dose received. The H460 and MCF7 cell line displayed a statistically significant ($\hbox {p}<0.005$) difference in mean PC1 score, for all radiation doses (2–10 Gy), relative to un-irradiated cells for days 1–3 (H460) and 2–3 (MCF7) post radiation. LNCaP cells did not display any significant change in mean PC1 score, regardless of dose or time (Fig. 2C). Despite all three cell lines exhibiting reductions in proliferation and increased cell death, with a dose dependent trend, clonogenic survival assays indicated that H460 and MCF7 cells are significantly more resistant to radiation than LNCaP cells.

In Fig. 2D the mean score corresponding to the glycogen basis spectrum used in the GBR-NMF model is shown, obtained for each cell type at each dose of radiation. This score plot shows the ability to replicate the plot derived from PC1 scores, using GBR-NMF modelling, with striking accuracy. In both cases, H460 and MCF7 cells displayed an upward trend toward glycogen expression in response to treatment with higher doses of radiation, as well as in response to the time at which analysis was carried out post treatment. The upregulation of glycogen is partly due to inactivation of glycogen synthase kinase 3$\beta$ (GSK-3$\beta$), an isoform of GSK-3, which is heavily involved in energy metabolism and has been implicated in radiation cytotoxicity responses^11,24^ in all three cell types when comparing irradiated samples with unirradiated controls.

### Monitoring glycogen and glucose levels

**Figure 3:**
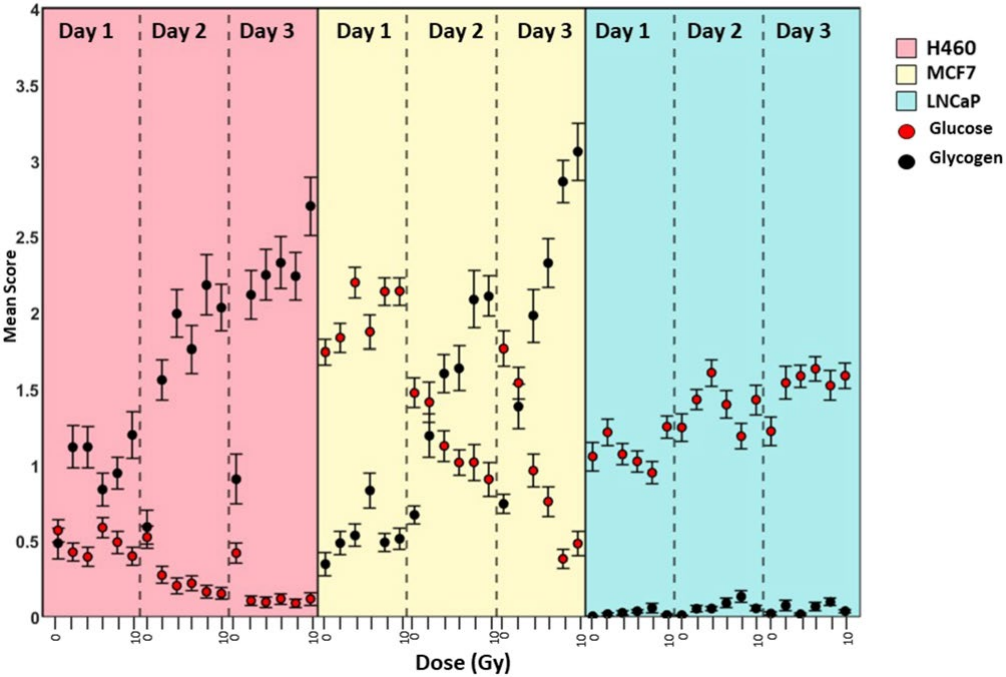
Mean scores for glycogen (black) and glucose (red) for H460 cells (pink), MCF7 cells (yellow) and LNCaP cells (blue) for doses 2–10 Gy on days 1–3 post irradiation, obtained using the GBR-NMF model. Error bars represent $+/-$ 1 standard error.

The GBR-NMF model was used to obtain scores on 30 chemical bases listed in Table S1. As an example of the ability to monitor interactions between various biochemicals using the GBR-NMF model, glycogen and glucose expression were plotted using the GBR-NMF scores on each of these chemical bases, for each cell line with respect to radiation dose and time of analysis following radiation exposure. The resulting boxplot is shown in Fig. 3 wherein the mean score for each sub-group is plotted for glycogen (black) and glucose (red). For both radio resistant cell types, H460 (pink) and MCF7(yellow), glucose scores appeared to decrease as the glycogen score increased. The linear correlation coefficient ($\hbox {p} <0.01$, $\alpha = 0.05$) of glucose and glycogen scores was $-0.56$, $-0.75$ and $-0.18$ for H460, MCF7 and LNCaP cells, respectively. As glycogen is formed from glucose monomers^25^, it was expected that an increase in glycogen score would result in a decrease in glucose score. An exception to this trend was observed in MCF7 cells the first day after radiation exposure, wherein glucose scores appeared significantly higher than glycogen scores for all doses including the unirradiated control. This observation was also true of the unirradiated controls and the cells which received a 2 Gy dose of RT across all days for MCF7 cells. In both days two and three following radiation exposure, the cells which received 2 Gy dose displayed more similar scores in glucose and glycogen expression. This suggests that treatment with doses of less than 4 Gy does not promote the same level of glycogen accumulation as seen with doses of 4 Gy and higher or that significant decrease in glucose levels does not occur for doses of 2 Gy or less within 1–3 days post irradiation. A similar trend was observed for H460 cells, in that mean glucose score decreased significantly as glycogen score increased across all doses. The relationship between glucose and glycogen expression was less linear in H460 cells when compared with MCF7 cells. This is also consistent with the much greater increase in glycogen score observed for H460 cells for all doses compared with unirradiated controls than was observed for MCF7 cells, which displayed a more linear relationship with glycogen expression and dose received. LNCaP cells did not display any significant decrease in glucose score with respect to radiation.

### Identification of radiation induced biochemical changes using GBR-NMF scores and random forest

**Figure 4:**
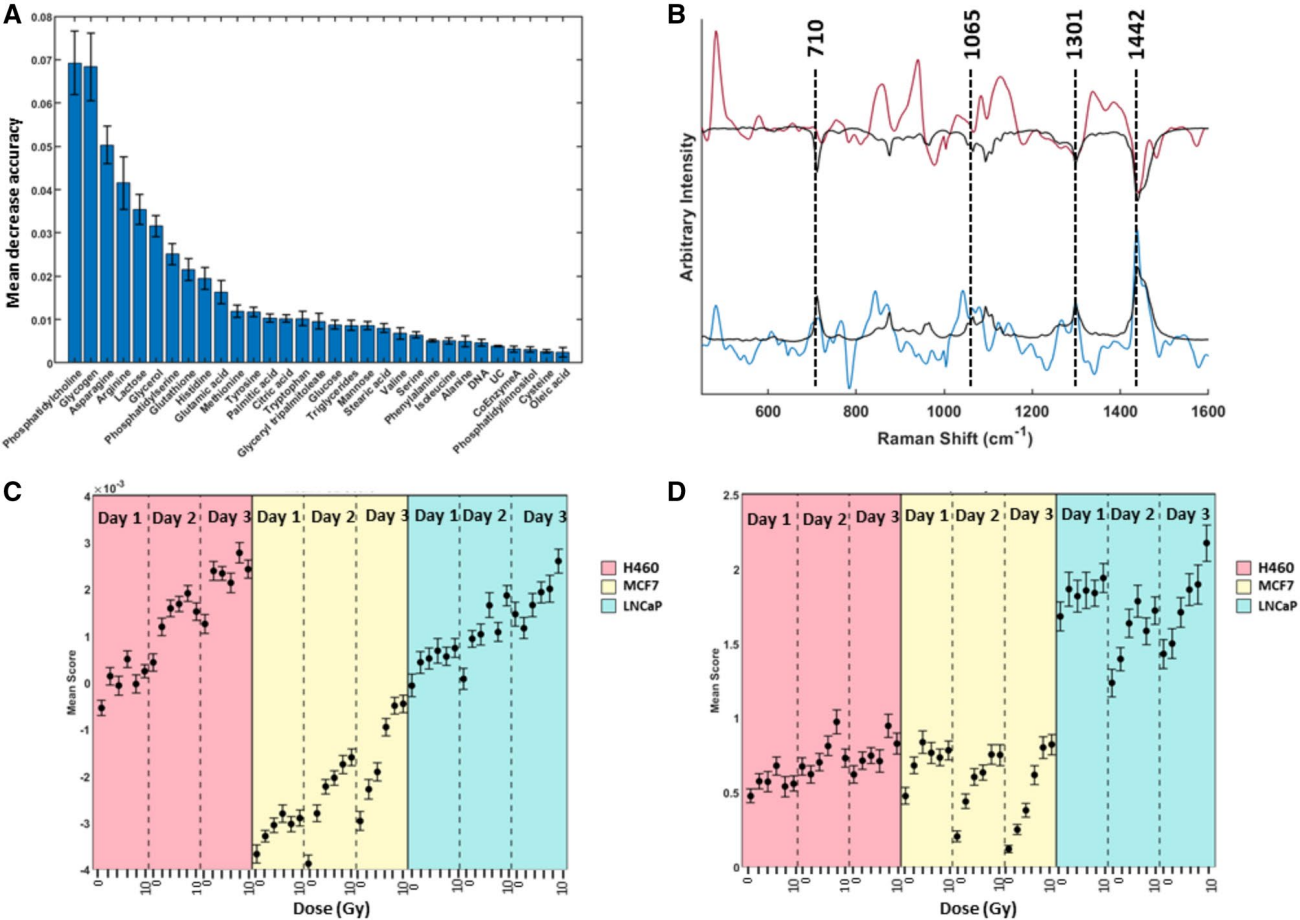
**(A)** Bar chart showing variable importance prediction obtained using GBR-NMF scores for 30 chemical bases plus 1 unconstrained component in a random forest decision model. The values shown are the average of 10 RF models using 75% of the original data for training. Error bars represent $+/-$ 1 standard deviation. **(B)** PC1 spectrum (red) and PC2 spectrum (blue) shown overlaid with pure phosphatidylcholine spectrum used for GBR-NMF modelling to show overlap of spectral features present in both PC1 and PC2 with phosphatidylcholine. Spectra have been scaled and offset for clarity. Phosphatidylcholine spectrum has been inverted in overlay with PC1 to highlight the similarities with the negative component of PC1. **(C)** Mean PC2 scores for H460 cells (pink), MCF7 cells (yellow) and LNCaP cells (blue) for doses 2–10 Gy on days 1–3 post irradiation. Error bars represent $+/-$ 1 standard error. **(D)** Mean phosphatidylcholine scores for H460 cells (pink), MCF7 cells (yellow) and LNCaP cells (blue) for doses 2–10 Gy on days 1–3 post irradiation. Error bars represent $+/-$ 1 standard error.

MDA values for each chemical base were calculated using a random decision forest consisting of 200 decision trees and the scores obtained on each of these chemical bases using the GBR-NMF model. The data input included all unirradiated controls, each dose and day for all three cell types. The data was then categorised as radio-resistant (H460 and MCF7) and radio-sensitive (LNCaP). The data was randomly split into a training set (75% of the original data) and a testing set (25% of the original data). The resultant MDA values for each of the 31 variables (30 chemical bases spectra and 1 unconstrained component) are shown in Fig 4A.

Analysis of the MDA of each variable revealed phosphatidylcholine to be the most important variable in correctly classifying the data as radiation resistant or radiation sensitive. This was unexpected as PCA analysis had shown glycogen to be a major biochemical which showed distinctly different expression patterns between radiation resistant and radiation sensitive cell types. However, it is shown in Fig 4A that glycogen contributes to a slightly lesser extent than phosphatidylcholine in terms of reducing the mean error associated with classifying the data using random forest. The five most important variables were identified as phosphatidylcholine, glycogen, asparagine, arginine and lactose. As phosphatidylcholine was identified as being the variable with the largest influence on the MDA of the overall RF model, the Raman spectrum used for this chemical base (black trace in Fig 4B) was overlaid with PC1 (red) and PC2 (blue), shown in Fig 4B. The overlay of phosphatidylcholine with PC1 and PC2 shows that both PC1 and PC2 contain spectral features which closely match those present in the phosphatidylcholine spectrum. PC1 has two closely matched peaks with phosphatidylcholine at $1301\,\hbox {cm}^{-1}$ and $1442\, \hbox {cm}^{-1}$ in the negative region of the spectrum. The overlaid phosphatidylcholine spectrum has been inverted for clarity. PC2 has similarities at $710 \,\hbox {cm}^{-1}$, $1065 \,\hbox {cm}^{-1}$, $1301 \,\hbox {cm}^{-1}$ and $1442 \,\hbox {cm}^{-1}$ with a small shoulder peak at $1453 \,\hbox {cm}^{-1}$ in the positive region of the spectrum. As the positive region of PC2 closely resembled the Raman spectrum of phosphatidylcholine, mean PC2 scores were compared with mean phosphatidylcholine scores from the GBR-NMF model, shown in Fig 4C,D, respectively.

**Figure 5:**
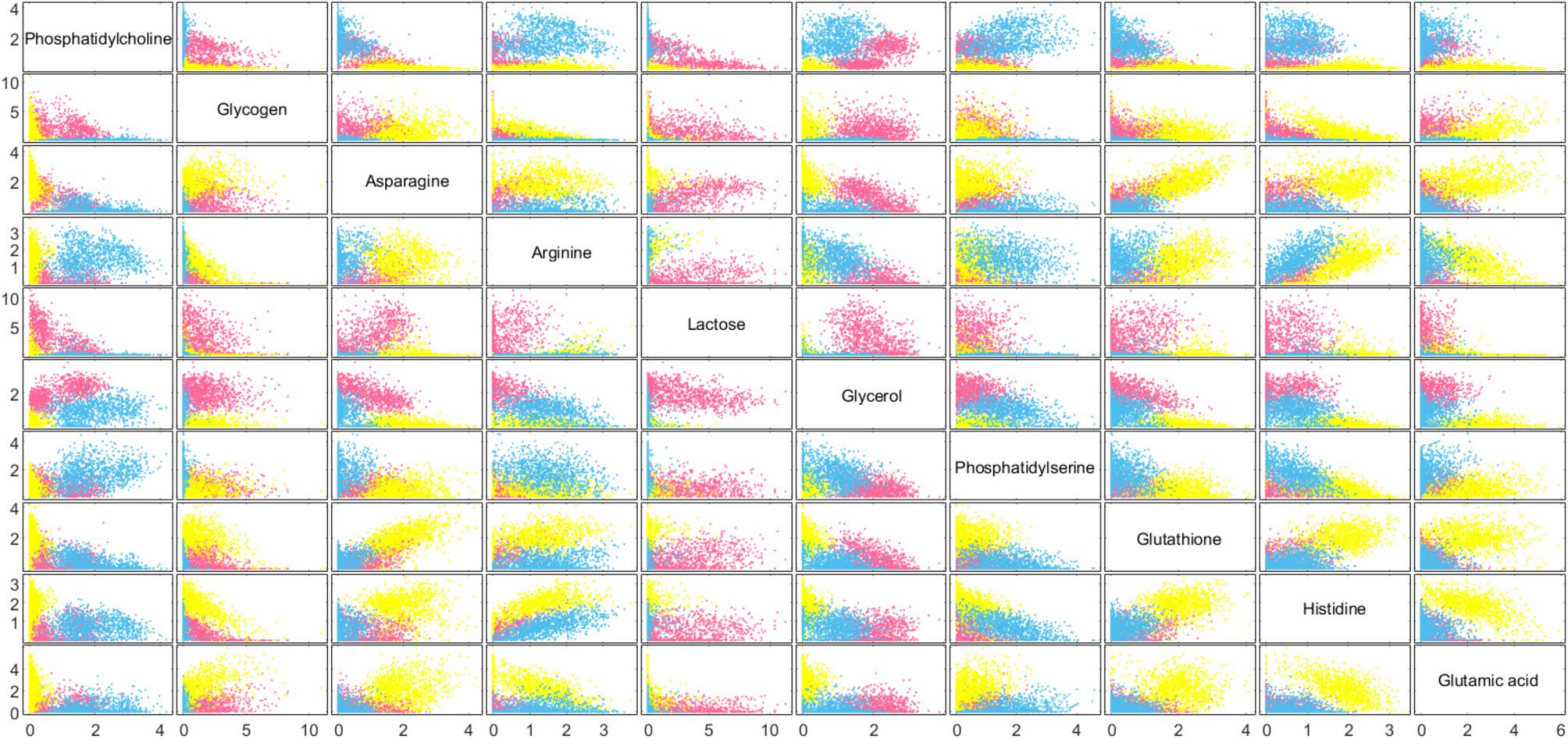
Scatter plot depicting scores for each cell line H460 (pink), MCF7 (yellow) and LNCaP (blue) on the 10 most important chemicals obtained from random forest decision modelling (Fig 4A), as labelled in the diagonal.

The GBR-NMF approach also allows us to visualise possible relationships between the input chemicals, for example, glucose and glycogen. The scores for each cell line, H460 (pink), MCF7 (yellow) and LNCaP (blue) from the ten most important chemicals (top 10 MDA scores in Fig 4A) in reducing the MDA value are shown as a scatter plot in Fig 5. The scatter plot shows clear separation between the three cell lines across the majority of the input chemicals. Additionally, this scatter plot highlights some of the differences in score trends across the three cell types. For example, H460 cells displayed a strong separation from the other two cell types when lactose was plotted against the remaining 9 top scoring biochemicals. Similarly, MCF7 displayed a distinctly different trend in score for glutamic acid, glutathione and asparagine. The most noticeable difference in score pattern for LNCaP cells compared with H460 and MCF7 cells was observed for glycogen, phosphatidylcholine and phosphatidylserine.

To discern whether the GBR-NMF model could reliably distinguish radio-sensitivity from radio-resistance, random forest was used to predict and classify the data based on GBR-NMF scores for the 30 chemical spectra listed in Table S1 and one unconstrained spectrum. The results from the prediction are shown in Table 1. The random forest algorithm correctly classified 1066 true positive values, as well as 548 true negative values. The misclassification rate was low, with only 5 false positives (0.3% of total dataset) and 0 false negatives. The accuracy, specificity and sensitivity were calculated and are listed in Table 2.

**Table 1:** Random forest prediction of radiosensitivity vs. radioresistance.

Observed	Prediction	
	Radiosensitive	Radioresistant
Radiosensitive	1066	0
Radioresistant	5	548

**Table 2:** Random forest prediction of radiosensitivity vs. radioresistance evaluation.

	Accuracy%	Sensitivity%	Specificity%
Radiosensitivity	99.8	99.5	100

### Identification of new radiation response profiles

**Figure 6:**
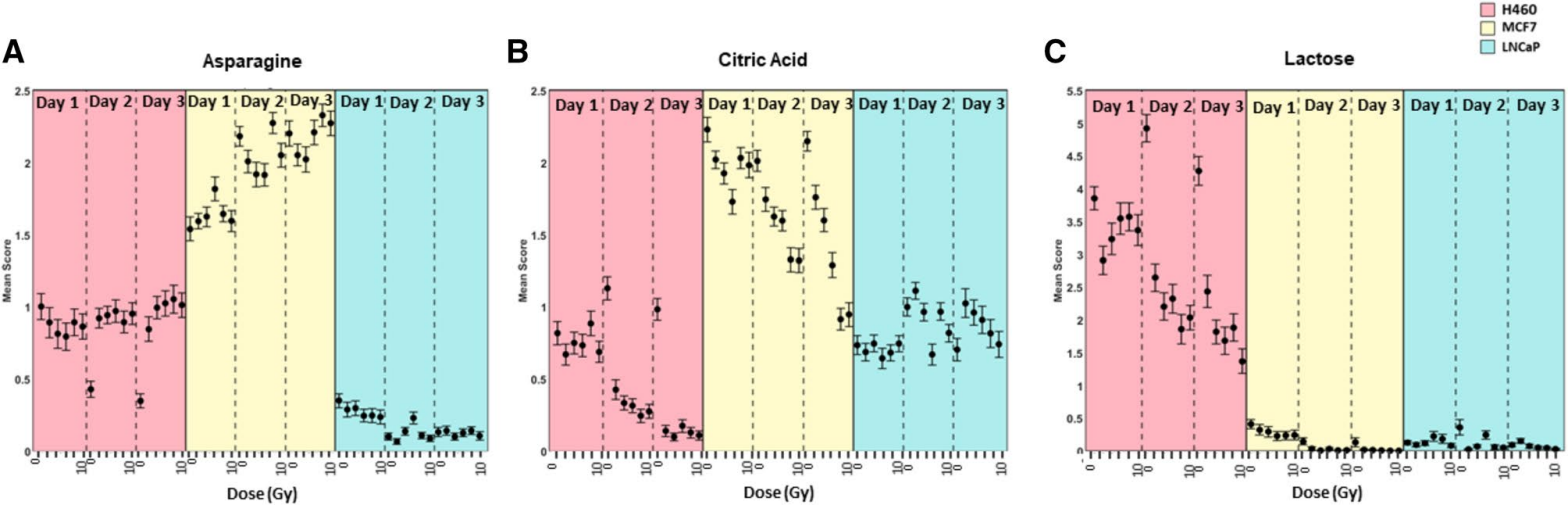
Mean scores obtained for H460 cells (pink), MCF7 cells (yellow) and LNCaP cells (blue) for doses 2–10 Gy on days 1–3 post irradiation for asparagine **(A)**, citric acid **(B)** and lactose **(C)**. Error bars represent $+/-$ 1 standard error.

The scatter plot matrix considered all doses and days combined for each cell type as three separate groups. To investigate whether any of the biochemicals which caused separation between the three groups displayed any trend with regards to radiation dose received, box plots of mean scores for asparagine, citric acid and lactose, with respect to dose and day were plotted for each cell type as shown in Fig. 6A–C, respectively. Figure 6A shows that the mean score for asparagine varies significantly across all three cell lines, irrespective of dose and day, which was expected from the scatter plot matrix shown in Fig. 5. The mean score on asparagine shows a trend with respect to radiation dose received for H460 cells. For days 1–3 the unirradiated control sample displayed a downward trend in mean asparagine score. However, for days 2 and 3 subsequent to radiation exposure, all doses (2–10 Gy) displayed a statistically significant difference ($\hbox {p}<0.001$) in mean asparagine score, when compared with the unirradiated control. This was not true of MCF7 and LNCaP cells. Conversely, mean scores of citric acid (Fig 6B) showed similar patterns across MCF7, H460 and LNCaP cell types. H460 cells displayed a statistically significant ($\hbox {p}<0.001$) decrease in mean score for doses 2–10 Gy when compared with the unirradiated controls on the same day. A similar trend was observed in mean score for MCF7 cells which exhibited a significant decrease in mean score for all days and doses when compared with unirradiated controls, with the exception of doses 8 and 10 Gy on day 1. LNCaP cells exhibited a significant change in mean score from unirradiated controls in only three cases (6 Gy on day 2 and 2 Gy and 4 Gy on day 3). The differences in trends noted between H460 and MCF7 cells with LNCaP cell types suggest the decrease in citric acid content with respect to radiation exposure may be linked to radiation resistance. Mean score plots for lactose are shown in Fig. 6C and similarly to asparagine, only H460 cell types displayed any significant change in mean score with respect to radiation dose received. In this case, a statistically significant change in mean score was observed for all doses (2–10 Gy) across all days when compared with unirradiated controls. Despite lactose being identified as an important variable in distinguishing between cell type (0 Gy controls) (Fig. S3), there appears to also be some correlation with decreasing lactose content and increased radiation exposure for H460 cells.

## Discussion

We have demonstrated, as proof of concept, the capabilities of a GBR-NMF model using a spectral library of 30 biochemicals as a method of identifying radiation response profiles across three different cancerous cell lines. Previously discovered PCA-based trends in glycogen expression due to radiation exposure were replicated using the GBR-NMF model. Matthews et al.^7^ highlighted the complexity of the metabolic pathways involved in radiation resistance, which calls attention to the fact that multiple metabolic processes can impact the levels of a single matabolite. This in turn can make it challenging to identify mechanisms which promote radiation resistance within multiple cell types and in turn potentially exploit such pathways to increase radiation sensitivity. The main advantage of using a GBR-NMF approach is that this model allows us to identify individual biochemicals which show increased or decreased expression as a result of both cell type and radiation exposure. To our knowledge, the most currently accepted method of identifying changes in metabolite expression is immuno histochemical (IHC) staining for metabolic enzymes, which is both time consuming and can often be subjective^26,27^. Another advantage of the GBR-NMF method is the distinct absence of negative features in the bases spectra, which can often be a problem when analysing PCA score trends relating to PCs with both positive and negative features. Additionally, as the model is semi-supervised and therefore each base is constrained to contain only one chemical spectrum, score trends can be directly related to the input chemical base. This is not often the case when PCA modelling is used as PCs often contain spectral features from multiple biochemicals, again making interpretation difficult and subjective^18^.

Glucose and glycogen scores were plotted for all three cell lines for unirradiated controls and doses 2–10 Gy on days 1, 2 and 3 post irradiation. The relationship between glucose and glycogen scores were as expected for each cell line^28^. Although not an entirely linear relationship, both H460 and MCF7 cells displayed decreasing glucose scores as glycogen scores increased, a pattern not observed for LNCaP cells. It is to be expected that glucose content would decrease with increasing glycogen content as glycogen is a polymer of glucose residues linked together by $\alpha -(1,4)-$glycosidic bonds. Interestingly, MCF7 cells displayed the highest glucose scores of the three cell types, with LNCaP cells also displaying high mean glucose scores across all conditions. This is not entirely unexpected as increased glucose uptake is a hallmark of cancer cells as noted by Otto Warburg in 1927^29^ and in several recent studies^25,30,31^. The difference in mean glucose scores in the 0 Gy controls across the three cell types could be due to many factors including cell type^30,32^ and aggressiveness of proliferation rates^33^.

Each of the biochemicals listed in Table S1 were investigated in order to determine any changes related to both radiation exposure and potentially radiation resistance within all three cell types. Random forest decision modelling showed that the most important variable in classifying the data as radio-resistant or radio-sensitive was phosphatidylcholine. Phosphatidylcholines are a class of phospholipids that incorporate choline as a head group and are a key component of membranes within eukaryotic cells^34^. This finding is supported by multiple theories which suggest the re-programming of lipid metabolism as an important feature in promoting malignant growth of cells^34–37^. Previous work in our group has used standard NMF algorithms to identify lipid like spectra as factors which displayed unique score patterns with respect to radio-sensitivity and dose of radiation received^38^. By constraining the bases in the ${\mathbb {S}}$ matrix, we here can move one step further by identifying the score patterns of specific lipids.

Identification of phosphatidylcholine as an important variable in contributing to the classification of spectra as belonging to a radio-sensitive or radio-resistant cell line also helped to identify many of the spectral features present in PC 2. This again highlights the benefit of GBR-NMF modelling over other dimensionality reduction techniques such as PCA as this information would likely have been overlooked or more difficult to discern from analysis of the PC components alone. The mean PC2 scores exhibited a very similar pattern to the mean phosphatidylcholine scores for both H460 and LNCaP cell types. MCF7 cells displayed a trend toward less negative PC2 scores with increasing dose of radiation and time post treatment. Therefore, it is unlikely that the MCF7 PCA score pattern pertains to that of phosphatidylcholine and more likely the negative features of PC2, which appear to mostly belong to nucleic acid ($784\, \hbox {cm}^{-1}$, $902 \,\hbox {cm}^{-1}$), phenylalanine ($1003\, \hbox {cm}^{-1}$) and Amide III and collagen ($1235 \,\hbox {cm}^{-1}$, $1334 \,\hbox {cm}^{-1}$)^39,40^. The reason for the differences in score plots for H460 and LNCaP cells can likely be attributed to the presence of various other spectral features within PC2 (Fig. 4B). Using GBR-NMF modelling allows us to assign scores to specific biochemicals that may contribute to identifying metabolic pathways in response to radiation treatment. An important point to note is that GBR-NMF allows us to distinguish scores from the positive and negative features of principal components separately. This is achieved by inclusion of individual spectra to which those features physically relate to within the library of chemical bases. For example, the PC2 scores of MCF7 cells only give us information on the negative component of the spectrum, positive PC features are not represented in the scores. The GBR-NMF model provides scoring information on both positive and negative features of the PC, provided each spectrum is included in the GBR-NMF model.

Classification of radio-sensitivity vs. radio-resistance was achieved with 99.8% accuracy using the scores on the 30 chemicals listed in Table S1 and the scores on one unconstrained component. The combination of GBR-NMF and random forest modelling identified phosphatidylcholine and glycogen as the two most distinguishing factors between the two radio-resistant and one radio-sensitive cell type investigated in this study, however there were also noticeable contributions from asparagine, citric acid and lactose.

H460 cells displayed a statistically significant increase in mean asparagine score for all doses compared with unirradiated controls for days 2 and 3 post treatment, which was not observed in the other two cell types. It is well documented that malignant cells generally exhibit increased amino acid uptake^41–44^. Asparagine has also been noted to promote amino acid uptake, particularly of serine and threonine^45^, which is favourable and prevalent in cancer cells due to the increased requirement for building blocks to support the increase in proliferation rates. Krall et al.^44^ provided evidence that asparagine may also act as an amino acid exchange factor in cancer cells, wherein intracellular asparagine exchanges with extracellular amino acids, in particular, serine, histidine and arginine, thus promoting increased cell proliferation and growth, however they did not investigate radiation effects on asparagine expression.

Both H460 (day 2–3) and MCF7 (day 1–3) cells exhibited a significant decrease in citric acid score compared with unirradiated controls on the same day. This finding implies that the radio-resistant cell types (H460 and MCF7) displayed a different pattern in mean score of citric acid when compared with radio-sensitive LNCaP cells. More recently, there has been significant interest in the citric acid cycle (TCA) and its potential as a therapeutic target for certain types of cancers^46^. Most early studies into cancer cell metabolism assumed that cancer cells bypass the TCA and utilise aerobic glycolysis as a primary energy source^47^. However, it is now generally accepted that some cancers rely heavily on the TCA for energy production^47^. The decreased citrate content in radio-resistant H460 and MCF7 could be an indicator of increased utilisation of the TCA as an energy source in response to ionising radiation.

As with asparagine, H460 cells were the only cell type to display a significant change in lactose score for all doses (2–10 Gy) when compared with unirradiated controls on the same day post treatment. Lactose was identified as an important variable in distinguishing between the three cell types using unirradiated controls. Therefore, it is possible that high lactose content is inherent to H460 cells, irrespective of radiation, however there appears to be a relationship with mean lactose score and radiation dose received by H460 cells (Fig. 6).

At present there is an unmet clinical need to identify factors that contribute to radiation response. Here we have demonstrated a proof of concept approach wherein a chemical base library can be exploited in order to obtain scores corresponding to each of these biochemicals for cells under various conditions, in our case radiation exposure. As future work, confirmation of the increased or decreased expression of the various biochemicals identified herein would be corroborated using IHC or liquid chromatography-mass spectrometry (LC–MS). Identification of radiation induced metabolic processes, which may ultimately lead to radio-resistance within certain cell types, has the potential to aid in the development of new combination therapies wherein radio-sensitising drugs can be used alongside radiation treatment and ultimately lead to better outcomes for patients undergoing radiation therapy.

## Methods

### Cell lines

H460 (ATCC# HTB-177), MCF7(ATCC# HTB-22) and LNCaP (ATCC# CRL-1740) cells were obtained as stock solutions from American Type Culture Collection (ATCC, Manassas, VA, USA). Cells were cultured as monolayers, at $37^{\circ }$C and 5% $\hbox {CO}_2$. Cells were cultured in RPMI 1640 (H460 and LNCaP) or DMEM (MCF7) as previously described^7^. All media components were purchased from Hyclone Laboratories Inc.(San Angelo, TX, USA).

### Irradiation

Cells were harvested and equivalent aliquots were incubated for 4 days at an initial cell density determined to achieve 50% confluency at time of irradiation. One hour prior to irradiation, culture media was replaced fresh media (SigmaAldrich Canada Co., Oakville, Canada). Cell monolayers were irradiated with a single fraction of 6 MV photons from a Varian 21 EX linear accelerator (Vairan Medical Systems, Palo Alto, CA, USA) at a dose rate 6 Gy/minute. Single fractions of 0, 2, 4, 6, 8, and 10 Gy were delivered to 3 cultures per dose.

### Raman spectroscopic acquisition and spectral processing

Cell preparation for Raman spectral acquisition and processing was performed as described previously^7^. Briefly, cells were washed with PBS, harvested with trypsin and centrifuged into a pellet. Pellets were transferred to a 5 mm thick magnesium fluoride window (Janos Technology Inc., Keene, NH, USA) and allowed to air dry for 5 min before spectral acquisition.

Raman spectra were acquired from 20 individual cells from each sample (20 spectra per sample at the radiation doses indicated) over 3 days of analysis performed in triplicate for all 3 cell lines. Cells for analysis were chosen at random from the top layer of the cell pellet. Spectral acquisition was performed with an inVia Raman microscope (Renishaw Inc., Gloucestershire, UK) with a 100X dry objective ($\hbox {NA} = 0.9$) (Leica Microsystems, Concord, Ontario, Canada), a 600 lines / mm diffraction grating, a 10 s acquisition time per cell and a 450–$1800\,\hbox {cm}^{-1}$ spectral window. Spectra were recorded with a thermoelectrically cooled iDus CCD detector (Andor Technology, Belfast, UK). A 785 nm laser (Renishaw) was used for excitation. The laser power density at the sample was 0.5mW /$\upmu \hbox {m}^3$ with a sampling volume of $2 \times 5 \times 10 \upmu \hbox {m}$ to allow single cell Raman spectrum acquisition. Each cell spectrum was processed to remove cosmic rays, correct for wavenumber calibration drifts, estimate and subtract a baseline arising from the substrate and biological fluorescence, and normalised such that the total area under the curve is equal to 1. The fully processed data sets were analysed with principal component analysis using standard algorithms in Matlab.

### Semi-supervised non-negative matrix factorisation and random forest modelling

Group and basis restricted non-negative matrix factorisation (GBR-NMF) was performed on the spectra in order to decompose the data matrix, ${\mathbb {X}}$, into three lower rank matrices such that ${\mathbb {X}} \approx \mathbb {WAS}$. These three matrices included the chemical bases responsible for variation in the spectra (${\mathbb {S}}$), a matrix responsible for scaling the bases (${\mathbb {A}}$), and the scores on the bases representing the contribution of each chemical to each spectrum (${\mathbb {W}}$). GBR-NMF modelling was carried out using R version x64 3.6.1. Random forest (RF) modelling was carried out on GBR-NMF scores obtained for the 30 chemical bases listed in Table S1 using the open source randomForest package in R (version x64 3.6.1). Data was split into a training (75% of the data) and testing set (25% of the data). The random forest was constructed using 200 decision trees and 5 randomly selected input variables were used to split each node. The testing dataset was then input to the random forest which was constructed using the training dataset, in order to obtain the predictions shown in Table 1. The out-of-bag (OOB) estimate of error obtained when the entire dataset was used to train the RF model was 0.3% and therefore no improvement on the split testing and training method (0.3% classification error), therefore we chose to split the data into training and testing subsets to allow for a more robust comparison with other classification methods in any future analyses. The hyperparameters used in the RF were compared with models consisting of no. of trees used ranging from 100–2000 (in increments of 100) and no. of variables used in split ranging from 1–30 (in increments of 1). The results of this comparison are displayed as a heat map of the corresponding OOB error (as a percentage, average of 5 models) using each combination of conditions in S4. This figure shows that very little performance difference existed between number of variables ranging from 4-8 and number of trees ranging from 200 to 1400.

## Supplementary information

Supplementary Information.

## Data Availability

Code for GBR-NMF is available on GitHub repository^48^.
