# Supplementary material for: Raman spectroscopy and group and basis-restricted non negative matrix factorisation identifies radiation induced metabolic changes in human cancer cells
Source: Sci Rep. 2021 Feb 16;11:3853. doi: 10.1038/s41598-021-83343-5 (PMC7886912; doi:10.1038/s41598-021-83343-5)
Supplement: Supplementary file 1 — Supplementary Information. [file 41598_2021_83343_MOESM1_ESM.pdf]

Supplementary Data - Raman Spectroscopy and Group and Basis-Restricted Non Negative Matrix Factorisation Identifies Radiation Induced Metabolic Changes in Human Cancer Cells

Kirsty Milligan[1], Xinchen Deng[1], Phillip Shreeves[2], Ramie Ali-Adeeb[1], Quinn Matthews[3], Alexandre Brolo[4], Julian J. Lum[5,6], Jeffrey L. Andrews[2], and Andrew Jirasek[1*]

[1]Department of Physics, The University of British Columbia, Kelowna, Canada
[2]Department of Statistics, The University of British Columbia, Kelowna, Canada
[3]BC Cancer, Centre for the North, Prince George, Canada
[4]Department of Chemistry, University of Victoria, Victoria, Canada
[5]Department of Biochemistry and Microbiology, University of Victoria, Victoria, Canada
[6]Trev and Joyce Deeley Research Centre, BC Cancer, Victoria, Canada
[*]andrew.jirasek@ubc.ca

Table S1. 30-reference-biochemical Raman chemical library

Alanine
Arginine
Asparagine
Citric acid
CoEnzymeA
Cysteine
DNA
Glucose
Glutamic acid
Glutathione
Glycerol
Glyceryl tripalmitoleate
Glycogen
Histidine
Isoleucine
Lactose
Mannose
Methionine
Oleic acid
Palmitic acid
Phenylalanine
Phosphatidylcholine
Phosphatidylserine
Phosphatidylinnositol
Serine
Stearic acid
Triglycerides
Tryptophan
Tyrosine
Valine

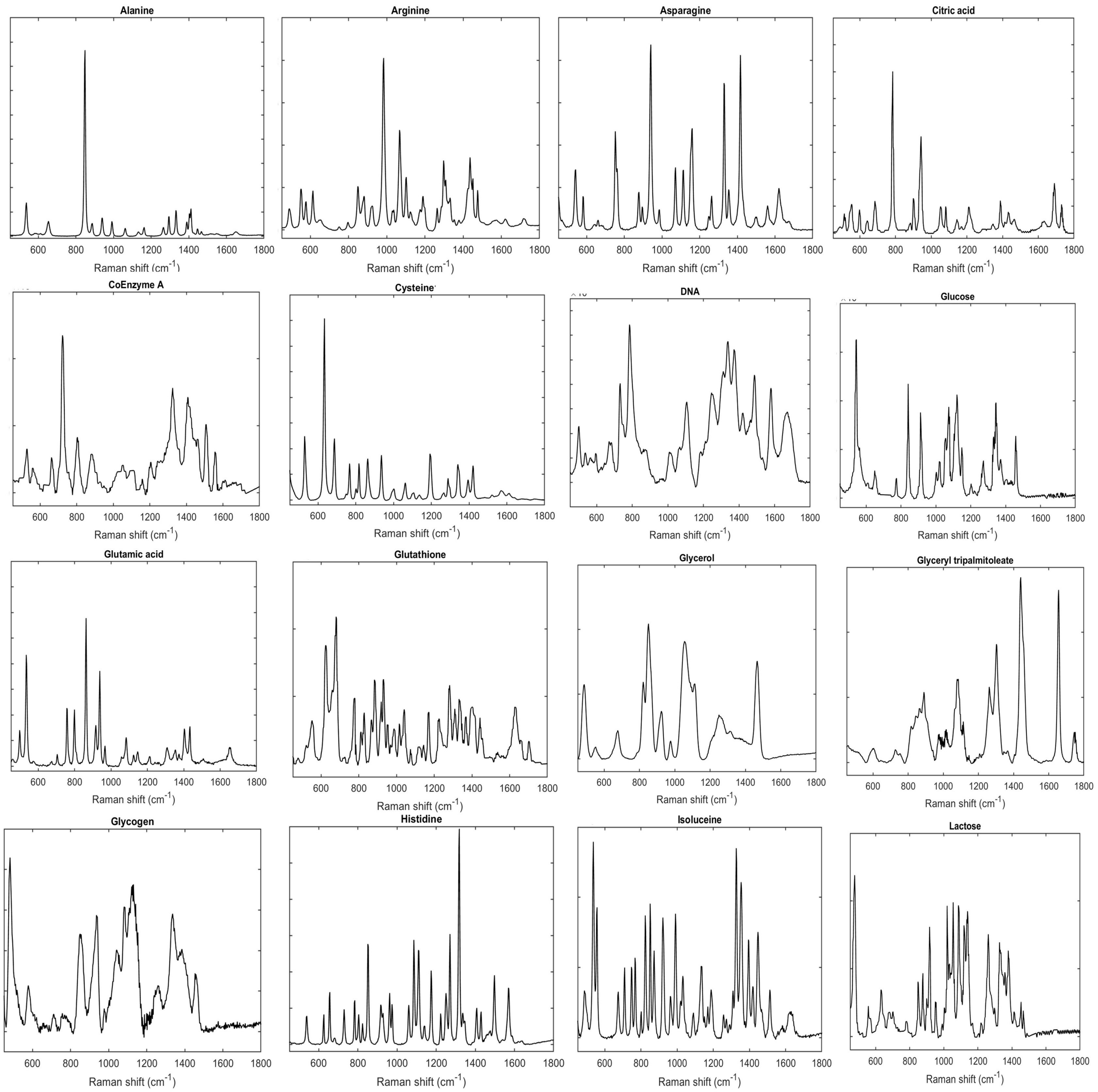

Figure S1. Raman spectra of biochemicals 1-16 as listed in table S1 which were used in the GBR-NMF model. All spectra were acquired using a Renishaw InVia system (dry 100x objective), using an excitation wavelength of 785 nm. Spectra were collected from biochemicals in their pure form. All spectra have been baseline corrected and smoothed using standard Savitsky-Golay filtering algorithms in MatLab. The spectra were then normalised such that the area under the curve was equal to 1.

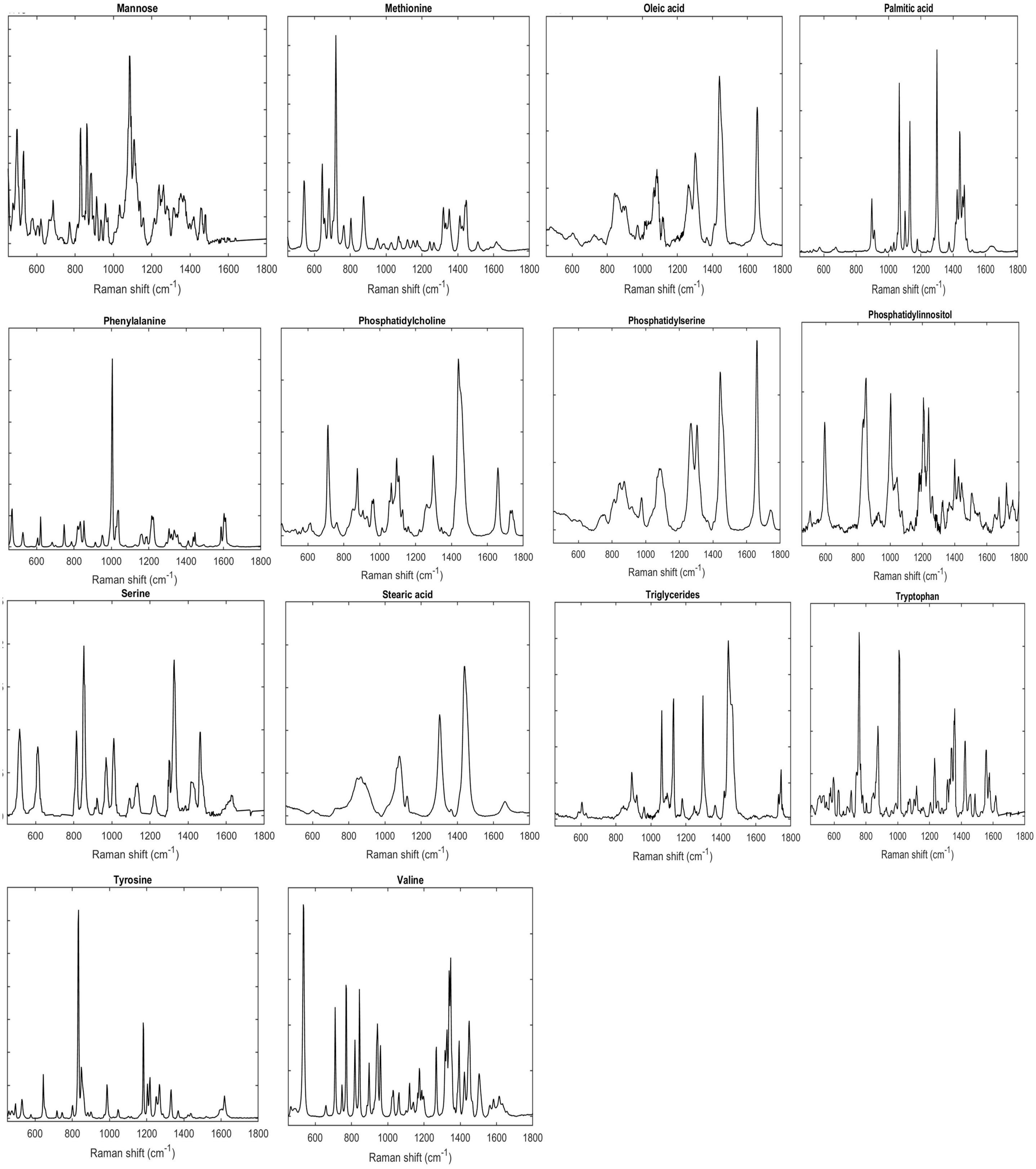

Figure S2. Raman spectra of biochemicals 17-30 as listed in table S1 which were used in the GBR-NMF model. All spectra were acquired using a Renishaw InVia system (dry 100x objective), using an excitation wavelength of 785 nm. Spectra were collected from biochemicals in their pure form. All spectra have been baseline corrected and smoothed using standard Savitsky-Golay filtering algorithms in MatLab. The spectra were then normalised such that the area under the curve was equal to 1.

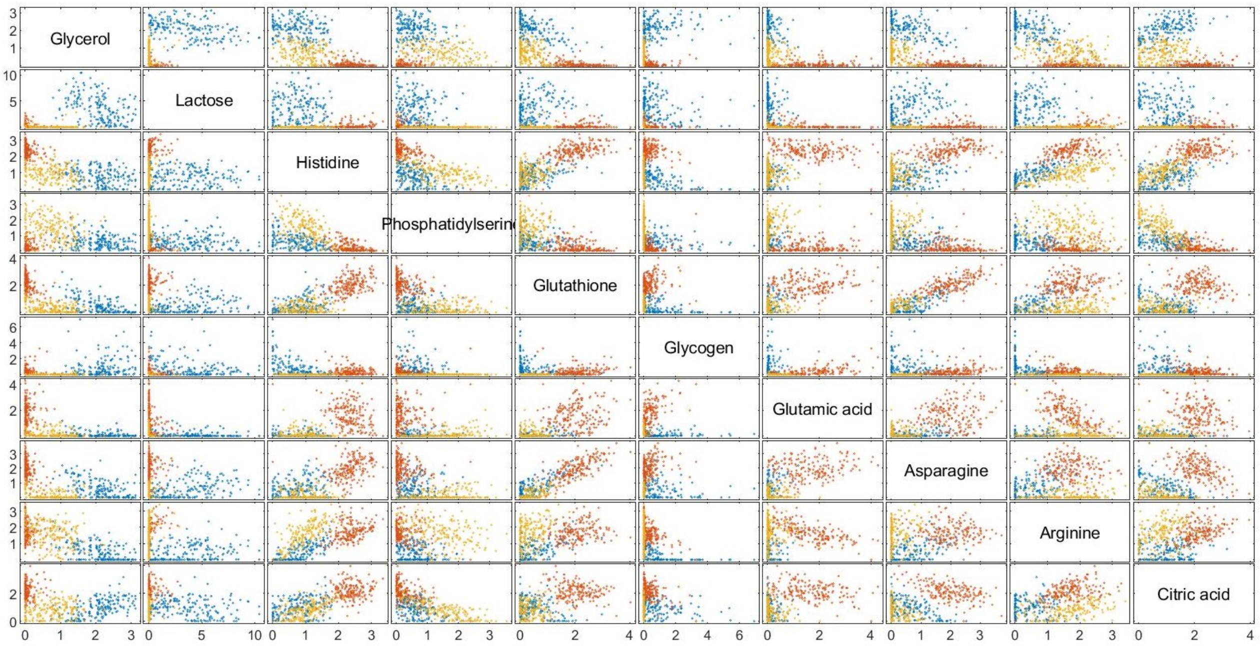

Figure S3. Scatter plot depicting scores of unirradiated controls for each cell line H460 (blue), MCF7 (red) and LNCaP (yellow) on the 10 most important chemicals obtained from random forest decision modelling, as labelled in the diagonal.

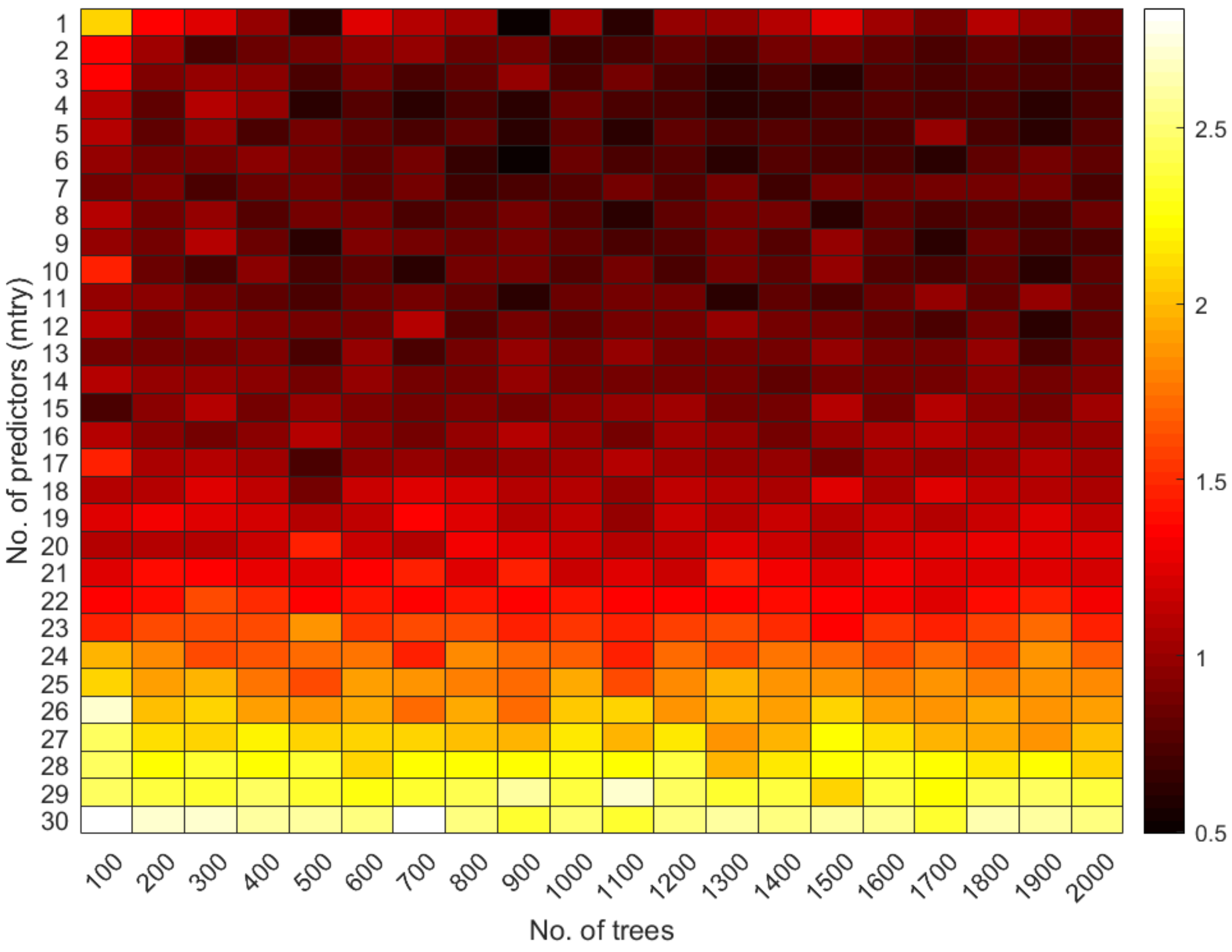

Figure S4. Heat map depicting OOB error as a misclassification percentage (average of 5 RF models) using various combinations of number of trees used in RF modelling (ranging from 100-2000 in increments of 100) and number of variables used to split each node (ranging from 1-30 in increments of 1) White pixels represent an error of 2.8% and deep red pixels represent an error of 0.5%. Colour bar depicts range in OOB error as a misclassification percentage. Heat map was generated using freely available software in Matlab version 2019b.
